# Non-ST Elevation Myocardial Infarction in Patients With Hypertensive Emergency

**DOI:** 10.7759/cureus.63783

**Published:** 2024-07-03

**Authors:** G. Thiruvikrama Prakash, Prafull Dhewle, Subash Chandra Bose, Vinodhkumar Kandibendla

**Affiliations:** 1 Department of Cardiology, E. S. Hospital, Villupuram, IND; 2 Department of Cardiology, Shrikrishna Hrudayalaya Hospital, Nagpur, IND; 3 Department of Cardiology, Government Medical College, Ananthapuramu, IND; 4 Department of Cardiology, Prakriya Hospitals, Nagasandra, IND

**Keywords:** acute myocardial infarction, non-st elevated myocardial infarction, hypertensive crises, diabetes, cardiovascular disease

## Abstract

Background

Hypertensive emergencies represent high-cardiovascular-risk situations defined by severe increases in blood pressure. The prevalence of hypertension in non-ST elevation myocardial infarction (NSTEMI) is higher compared to STEMI and there is a lack of studies on NSTEMI patients with hypertensive emergencies. Patients with diabetes exhibited a higher rate of hypertensive emergencies. This study's primary aim was to investigate the coronary artery disease profile in hypertensive emergency patients with NSTEMI, and the secondary aim was to determine the impact of diabetes on the development of hypertensive emergencies.

Methodology

A total of 100 patients with NSTEMI and hypertensive emergency presenting to the hospital were enrolled in the study. The duration of the study was 24 months. The patients were also sub-grouped into diabetic and nondiabetic. Baseline characteristics were noted, and coronary angiogram and renal angiogram were also done. Based on variables, the chi-square test and t-test were employed to assess the significance. *P*-value < 0.05 was considered statistically significant.

Results

The mean age at presentation for patients with NSTEMI and hypertensive emergency was 58 years. Patients consuming alcohol were slightly higher (28, 28%) than those who smoked (23, 23%). Among all, 48 (48%) patients had diabetes. When considering the number of vessels, diabetic patients had more single-vessel diseases (18, 37.5%) and nondiabetic patients had more double-vessel diseases (15, 28.8%). The mean ejection fraction of the diabetic group was 56.1% ± 6.8% and the nondiabetic group was 54.2% ± 7.7%. Among all the patients, 52 (62.6%) used combination drugs, while 39 (46.9%) were on defaulter drugs.

Conclusions

Several risk factors like age, smoking, alcohol, and nonadherence to drugs were found to have an association with the occurrence of hypertensive emergency. Diabetes was found to be significantly associated with unfavorable coronary anatomy among the population.

## Introduction

Persistent elevation of blood pressure in the arteries is a well-recognized risk factor contributing to atherosclerosis development [1]. The elevated force exerted by blood against artery walls, known as hypertension, can often go unnoticed without causing apparent symptoms, leading to an incidental diagnosis. However, in some instances, the persistent and uncontrolled nature of high blood pressure can manifest through chronic impairment of various bodily organs. In extreme cases, a hypertensive crisis may occur, characterized by a sudden and severe increase in blood pressure levels. This acute hypertensive state poses an immediate threat, as it directly inflicts damage to vital organs due to the excessive pressure within the vascular system. This mostly occurs in persons with preexisting hypertension.

Although the availability of safe, efficient, and well-tolerated antihypertensive drugs, the prevalence remains unchanged at about 0.2% [2-5]. On the other hand, in cases of non-ST elevation myocardial infarction (NSTEMI), the presence of hypertension stands as an independent risk factor that increases the likelihood of significant adverse cardiac events, both in the short and long term. However, when it comes to the long-term prognostic implications of hypertension in patients suffering from acute coronary syndromes, which encompass ST-elevation myocardial infarction (STEMI), NSTEMI, and unstable angina, the available data remains inconclusive [6]. Extensive population-based studies conducted on individuals diagnosed with NSTEMI have revealed that persistently elevated blood pressure, a condition known as chronic hypertension, emerges as the most commonly occurring risk factor. This cardiovascular risk factor is present in nearly two-thirds of the overall NSTEMI patient population investigated in these epidemiological research endeavors [7]. This higher prevalence of hypertension in NSTEMI concerning patients with STEMI (about 70%-75% versus 30%-40%) could be the reason that patients with NSTEMI are usually older and affected by more comorbidities with respect to patients with STEMI [8].

Despite divergent findings across different research studies, a large-scale investigation involving a cohort of approximately 6,000 participants demonstrated that individuals diagnosed with NSTEMI exhibited a higher mortality rate over two years when compared to those who experienced STEMI. This outcome contradicted the general expectations and highlighted the potential severity of NSTEMI, which is sometimes perceived as a less severe form of heart attack [8]. However, this has not been studied in patients with a hypertensive emergency [9]. Patients with diabetes exhibited higher rates of hypertensive emergency, particularly left ventricular failure [10].

There is a lack of data on hypertensive emergency patients with NSTEMI and clinical outcomes of hypertensive emergencies in both diabetics and nondiabetics. Thus, we aimed to study the coronary artery disease profile in hypertensive emergency patients with NSTEMI. Also, to evaluate the influence of diabetes mellitus on the occurrence of hypertensive emergencies by comparing the clinical and demographic profiles of patients who experienced hypertensive emergencies, categorized into those with and without a preexisting diagnosis of diabetes. This comparative analysis will shed light on the potential role of diabetes as a contributing factor in the development of these acute and life-threatening elevations in blood pressure.

## Materials and methods

This single-center, hospital-based, prospective observational study was conducted over 24 months, enrolling 100 patients with hypertensive emergencies and NSTEMI. The patients were further divided into two groups: diabetic and nondiabetic. Patients presenting with hypertensive emergency having NSTEMI were defined by positive cardiac biomarkers with either typical angina or electrocardiogram (ECG) changes or new regional wall motion abnormalities were included in the study. Patients with preexisting STEMI, pulmonary embolism, chronic obstructive pulmonary disease with cor pulmonale, sepsis, chronic kidney disease (GFR < 20 mL/1.73 m²), coronary artery disease, recent hospitalization for heart failure (<3 months), or any severe illness were excluded. The study was conducted after receiving approval from the ethics committee and per the Declaration of Helsinki. Informed consent was obtained from all study participants.

Data collection

A detailed history, including comorbidities and clinical examination, was collected. Baseline blood pressure and routine blood investigation such as complete blood count, renal function test, lipid profile, and cardiac biomarkers were recorded. Various imaging techniques like echocardiogram and ECG were performed for all the enrolled patients. A coronary angiogram was performed after obtaining informed consent, and a renal angiogram was conducted in select patients where indications were present. The study participants underwent a diagnostic procedure known as coronary angiography, which involved selectively inserting catheters into the left and right coronary arteries using standard techniques. Additionally, a separate procedure, called renal arteriography, was performed to visualize the renal arteries. For this purpose, a specialized catheter (Judkins right catheter) was sequentially introduced into the openings (ostia) of both renal arteries while positioning the patient in a left anterior oblique projection with an angulation of 10-20 degrees. Once the catheter was in place, an iodine-based contrast medium with an iso-osmolar concentration was administered, with a dose range of 5-10 mL injected into each renal artery. This allowed for detailed imaging and assessment of the renal vasculature. Significant coronary artery disease was defined as a narrowing or obstruction of the lumen (inner diameter) by more than 70% in at least one major coronary artery with a diameter of 2.5 mm or larger, observed from two different imaging angles. Additionally, a luminal obstruction of 50% or more in the left main coronary artery (LMCA) was also considered significant. Regarding the renal arteries, patients were classified as having significant renal artery stenosis if there was a narrowing of 60% or more in the lumen of one or both main renal arteries (including any accessory renal arteries) or one of their major branches. Patients were followed up until discharge, with monitoring every 24 hours until a coronary angiogram was performed. The average follow-up period was 72 hours.

Statistical analysis

Data were analyzed using Stats-direct software, version 2.7.9 (StatsDirect, Cheshire, UK). The researchers employed descriptive statistics to analyze the study sample. Categorical variables were summarized using numbers and percentages, while continuous variables were represented by their means and standard deviations. To compare the variables between the two groups of hypertensive patients, those with diabetes and those without, appropriate statistical tests were utilized. Specifically, the chi-square test was applied to evaluate the differences in categorical variables, while the *t*-test was employed to assess the significance of differences in continuous variables between the two groups. The normality assumptions of numerical data were assessed by the Shapiro-Wilk test.

## Results

Baseline characteristics of patients are detailed in Table 1.

**Table 1:** Baseline characteristics Values are presented as percentage or mean ± standard deviation (SD). *P* ≤ 0.05 was statistically significant. HDL, high-density lipoprotein; LDL, low-density lipoprotein; T2DM, type 2 diabetes mellitus

Variables	*N* = 100 patients
Age (years, mean)	58
Male (%)	78
T2DM (%)	48
Hypothyroidism (%)	5
Left ventricular hypertrophy (%)	64
Dyslipidemia (%)	28
Total cholesterol (mg/dL, mean ± SD)	173.1 ± 20.6
HDL (mg/dL, mean ± SD)	36.7 ± 7.8
LDL (mg/dL, mean ± SD)	113.6 ± 19.6
Alcohol (%)	28
Smoker (%)	23
Previous stroke (%)	4
Presentation BP (mmHg, mean)	192/122
Presenting symptoms	
Chest pain: Typical (%)	70
Chest pain: Atypical (%)	30
Creatinine (mg/dL, mean ± SD)	0.9 ± 0.2
Troponin T (ng/mL, mean ± SD)	0.6 ± 0.6
Hemoglobin (g/dL, mean ± SD)	12.5 ± 1.9
Ejection fraction (%, mean ± SD)	55.1 ± 7.3

Among the 100 patients, 48 (48%) of the patients had diabetes. Among all the patients 78 (78%) of the patients were male. Most of the patients had typical chest pain (70, 70%) compared to atypical chest pain (30, 30%). The average age of the study population was 58 years. The youngest among all the patients was of 39 years, and the eldest age was 81 years. In this study, 64 patients (64%) had left ventricular hypertrophy, while a minor percentage of patients had a history of hypothyroidism (5, 5%) and stroke (4, 4%). Patients consuming alcohol were slightly higher (28, 28%) than smokers (23, 23%). Table 2 shows the frequencies of antihypertensive drugs consumed by patients. Among all the patients, 52 (62.6%) used combination drugs and 39 (46.9%) had defaulter drugs. The most used among the patients was angiotensin receptor blocker (ARB; 30, 36.1%) followed by angiotensin-converting enzyme (ACE) inhibitor and diuretics (22, 26.5%). Alpha-blocker was the least-used drug among the study patients (3, 3.6%). To determine the impact of diabetes on patients with hypertensive emergency and NSTEMI, they were subdivided into two groups: diabetic and nondiabetic.

**Table 2:** Drugs for hypertension. Values are presented as number (%). ACE, angiotensin-converting enzyme; ARB, angiotensin receptor blocker

Hypertensive drugs	Frequency (*N* = 83 patients)
ACE inhibitor, *n* (%)	22 (26.5)
ARB, *n* (%)	30 (36.1)
Diuretics, *n* (%)	22 (26.5)
Thiazide, *n* (%)	18 (81.8)
Loop diuretics, *n* (%)	4 (18.2)
Beta-blocker, *n* (%)	12 (14.4)
Alpha-blocker, *n* (%)	3 (3.6)
Combination drugs, *n* (%)	52 (62.6)
Drug defaulter, *n* (%)	39 (46.9)

Baseline and angiographic details of hypertensive emergency patients with and without diabetes are summarized in Tables 3-4.

**Table 3:** Comparison of diabetic and nondiabetic patients with hypertensive emergency and NSTEMI. Values are presented as numbers (%). *P* ≤ 0.05 was statistically significant. CAG, coronary artery angiography; DVD, double-vessel disease; LAD, left anterior descending artery; LCX, left circumflex artery; RCA, right coronary artery; SVD, single-vessel disease; TVD, triple-vessel disease

Variables	Diabetes N= 48 patients	Non-Diabetes N=52 patients	Total N=100 patients	P value	Chi square value
Male, n (%)	33 (68.7)	44 (84.6)	77 (77)	0.099	2.727
Dyslipidaemia, n (%)					
Yes	20 (41.6)	7 (13.9)	27 (27)	0.008	10.191
No	28 (58.3)	45 (86.5)	73 (73)		
LAD, n (%)					
Yes	30 (62.5)	26 (50)	56 (56)	0.37	2.336
No	18 (37.5)	26 (50)	44 (44)		
LCX, n (%)					
Yes	26 (54.1)	18 (34.6)	44 (44)	0.126	1.628
No	22 (45.8)	34 (65.3)	74 (74)		
RCA, n (%)					
Yes	22 (45.8)	13 (25)	35 (35)	0.202	15.871
No	26 (54.2)	39 (75)	65 (65)		
No. of vessel disease, n (%)					
Normal	7 (14.5)	19 (36.5)	26 (26)	0.026	5.419
LAD	9 (18.7)	6 (11.5)	15 (15)		
LCX	9 (18.7)	3 (5.7)	12 (12)		
LAD + LCX	2 (4.2)	10 (19.2)	12 (12)		
RCA	0 (0.0)	3 (5.7)	3 (3)		
LAD + RCA	7 (14.6)	5 (9.6)	12 (12)		
LCX + RCA	3 (6.2)	0 (0.0)	3 (3)		
LAD + LCX + RCA	12 (25)	5 (9.6)	17 (17)		
CAG, n (%)					
Normal	7 (15.2)	19 (36.5)	26 (26)	0.144	-
SVD	18 (37.5)	13 (25)	30 (30)		
DVD	12 (25)	15 (28.8)	27 (27)		
TVD	11 (22.9)	5 (9.6)	17 (17)		

**Table 4:** Baseline characteristics of patients with diabetes and without diabetes in hypertensive emergencies with NSTEMI. Values are presented as mean ± standard deviation. *P* ≤ 0.05 was statistically significant. NSTEMI, non-ST elevation myocardial infarction

Variables	Diabetes	Without diabetes	Mean difference	Std. error difference	*P*-value	*T*-value
Age (years, mean ± SD)	57.4 ± 10.2	59.5 ± 8.4	-2.10	2.24	0.352	-0.938
Creatinine (mg/dL, mean ± SD)	0.92 ± 0.25	0.93 ± 0.18	-0.01	0.05	0.833	-0.212
Troponin T (ng/mL, mean ± SD)	0.54 ± 0.53	0.57 ± 0.72	-0.03	0.15	0.825	-.0.222
Hemoglobin (g/dL, mean ± SD)	12.47 ± 2.35	12.44 ± 1.48	0.02	0.47	0.962	0.047
Ejection fraction (%, mean ± SD)	56.1 ± 6.8	54.2 ± 7.7	1.89	1.77	0.287	1.072

The nondiabetic patients were older than diabetic patients (mean age 59.5 vs. 57.4 years). Most of the patients were male (33, 68.7% of diabetics and 44, 84.6% of nondiabetics), which was not statistically significant (*P* = 0.099). Most of the patients had dyslipidemia in the diabetic group (20, 41.6%) compared to nondiabetic (7, 13.9%). This finding was statistically significant (*P* = 0.008) in developing the hypertensive emergency. A total of 26 patients (26%) had normal coronaries, with a mean age of 62 years at presentation. In the nondiabetic patients, normal coronaries were found in 19 (36.5%) patients, and in diabetic patients, 7 (14.5%) had normal coronaries. This findings were clinically significant (*P* = 0.026). The Thrombolysis in Myocardial Infarction (TIMI) risk score for NSTEMI in hypertensive emergency was evaluated, and 70 (70%) patients had a TIMI score of 2 or 3. Among all 35 patients who underwent renal angiography, 3 patients had renal artery stenosis and all 3 of them underwent renal artery stenting. When considering the number of vessels, diabetic patients had more single-vessel disease (18, 37.5%) and nondiabetic patients had more double-vessel disease (15, 28.8%). The most involved vessel in both the groups was left anterior descending artery (LAD). The troponin T level was observed to be slightly higher in nondiabetic compared to diabetic patients (mean troponin T 0.57 ng/mL vs. 0.54 ng/mL). The mean ejection fraction of the diabetic group was 56.1 ± 6.8% and nondiabetic was 54.2 ± 7.7%.

## Discussion

A hypertensive crisis is a sudden, severe increase in blood pressure (180/120 mmHg). It is subdivided into two types, i.e., emergency and urgency. The mortality rate of hypertensive emergency is higher when compared with hypertensive urgencies [11]. So, our study focused on patients with hypertensive emergencies having NSTEMI. The enrolled patients were subgrouped based on diabetes and nondiabetes. A study done by Martin et al. reported that 23.4% of patients with hypertensive emergency had diabetes [12]. According to research conducted by Salagre et al., a substantial proportion, nearly 40%, of the patients experiencing hypertensive emergencies had a preexisting diagnosis of diabetes mellitus [13]. While numerical discrepancies exist, the study's findings unequivocally highlight the significant role played by the coexistence of diabetes and severely elevated blood pressure in precipitating hypertensive emergencies. This observation reinforces the established connection between diabetes mellitus and the development of critically high blood pressure levels, underscoring the intricate interplay between these two medical conditions. Metabolic abnormalities that accompany diabetes impair endothelium-dependent vasodilation [14], influence the pathological processes involved in the onset, and challenge the effective regulation of elevated blood pressure levels. Similarly, in our study, we observed 48 (48%) patients with hypertensive emergency having NSTEMI had diabetes. The mean age of the patients in our study was 58 years. Similarly, the study done by Pacheco et al. observed the mean age to be 62.6 ± 12.7 years in patients with a hypertensive emergency [15]. A study done by Al Bannay and Husain reported that age above 65 years and hypertensive emergency were predictors of mortality [10]. In our study, there was no significant difference in age among both groups (diabetes 57.4 ± 10.2 years, nondiabetes; 59.5 ± 8.4 years). However, in the study done by Al Bannay and Husain and Benenson et al., it was observed that diabetic patients were older in comparison with nondiabetic. The male preponderance was observed in our study among both groups. A systematic review by Talle et al. showed male predominance in 12 studies, while females predominated in 4 studies [9]. Among all the patients, 48 (48%) had diabetes, 28 (28%) had dyslipidemia, 28 (28%) consumed alcohol, and 23 (23%) had a history of smoking. Balahura et al. in their study mentioned that smoking, obesity, hypercholesterolemia, and diabetes were the most common predisposing factors leading to hypertension emergency [16]. Developing nations appear to experience a higher incidence of hypertensive emergencies, which may be attributable to inadequate management and control of the underlying risk factors contributing to such critical elevations in blood pressure. Among all, 39 patients (46.9%) in our study were drug defaulters. Elliott mentioned that probably the most important aspect of the treatment of a hypertensive emergency was to assure adherence to antihypertensive therapy [17]. A prospective research study revealed that among the factors examined, nonadherence to prescribed blood pressure medications emerged as the strongest predictor for the development of hypertensive emergencies in the study patients [18]. According to Balahura et al., low adherence and discontinuation of drugs were one of the common risk factors for developing hypertensive emergency [16]. Benenson et al. identified several risk factors for hypertensive emergency in diabetic patients. They observed that older age, nonadherence to medication, and the presence of comorbid conditions were associated with the occurrence of hypertensive emergency [19].

Literature findings indicated that diabetic patients with lower hemoglobin levels, a condition known as anemia, were at an increased risk of experiencing hypertensive emergencies. Anemia greatly contributes to renal ischemia and an increase in renal sympathetic activity. Sustained sympathetic activation upregulates the renin-angiotensin-aldosterone cascade, giving rise to blood pressure and likely predisposing to the development of hypertensive emergency. In diabetic patients with kidney disease, anemia may arise due to a deficiency in erythropoietin, a hormone responsible for red blood cell production, or a decreased responsiveness to this hormone, known as erythropoietin resistance [19]. In our study, both groups had normal hemoglobin values (diabetic 12.47 ± 2.35 g/dL vs. nondiabetic 12.44 ± 1.48 g/dL). In our study, three patients had renal artery stenosis. Renal artery stenosis is said to be significant with hemodynamic reperfusions, thus causing an imbalance of the renin-angiotensin-aldosterone cascade, resulting in accelerated hypertension [20].

Troponin T was elevated among both groups (diabetes 0.54 ± 0.53 ng/dL vs. nondiabetes 0.57 ± 0.72 ng/dL). An investigation that involved a retrospective analysis of data from patients who experienced hypertensive emergencies revealed that individuals with elevated levels of cardiac troponin, a biomarker indicative of heart muscle damage, faced a nearly threefold increase in the risk of experiencing cardiovascular events compared to those with normal troponin levels [21]. The most recent guidelines issued by the International Society of Hypertension advocate for the inclusion of a cardiac troponin assay as a crucial diagnostic test in the initial evaluation and workup of patients presenting with a hypertensive emergency [22].

In our study, we found that LAD was the most involved vessel (56, 56%) among both groups. Similarly, in one of the studies where patients with NSTEMI were evaluated, the high culprit lesion was in LAD (38%). It is said that patients with NSTEMI were least likely to present with a left circumflex artery (LCX) culprit lesion. One potential explanation is that the geometry of the LCX and its branches results in a difference in wall shear stress compared with LAD [23]. In our study, 44% of the patients had involvement of the LCX among all the patients. Previous literature has observed that the presence of multivessel disease, characterized by significant obstructions in multiple coronary arteries as seen through coronary angiography, was linked to unfavorable outcomes in hypertensive patients [24]. Konstantinou et al. stated that patients with either preexisting hypertension or diabetes or both display more often multi-vessel disease [25]. Contrast results were found in our study as single-vessel disease was high in the diabetic group (37.5%).

This was an observational study involving a small sample size. Larger prospective studies with larger cohorts were required to validate these findings. However, there was a lack of long-term follow-up and larger datasets to determine outcomes and draw further conclusions. Data on patients presenting with pulmonary edema and those with new-onset heart failure were not collected during the study.

## Conclusions

In conclusion, our study identified several risk factors like older age, smoking, alcohol, and nonadherence to medication for hypertensive emergencies in the population with NSTEMI. Although anemia is a high-risk factor in this population, no anemic condition was observed among the population. Multi-vessel involvement and total occlusion were lower in patients presenting with NSTEMI and hypertensive emergency. Diabetes among patients with hypertensive emergency having NSTEMI was found to be significantly associated with unfavorable coronary anatomy. LAD vessel involvement was observed more in both diabetic and nondiabetic groups.
